# A model for grid cells where spatially correlated place cells compete for the grid map nodes

**DOI:** 10.1186/1471-2202-14-S1-P2

**Published:** 2013-07-08

**Authors:** Luísa Castro, Paulo Aguiar

**Affiliations:** 1 Departamento de Matemática, Faculdade de Ciências da Universidade do Porto, Porto, Portugal; 2 Centro de Matemática da Universidade do Porto, Porto, Portugal

## 

Grid cells in the medial entorhinal cortex (mEC) encode space in a particular way: their firing rate intensity forms an equilateral triangular lattice as the animal moves in the environment [1]. Models addressing the generation of grid fields fall into two major classes, both with important limitations [2]. Most models based on interference of oscillations are not robust to noise and suffer from the prerequisite of two independent and stable network oscillations with similar frequency. Models based on recurrent networks, where the grid pattern emerges as a stable state of the network, suffer from the topographic assumption and the need for dense recurrent connections between mEC cells, which are both not supported by experimental data.

Here we present a novel firing rate model for the generation of grid fields with two very important features: i) grid fields are built on the spatial information provided by place cells, and ii) plasticity on the synapses between place cells and grid cells depend on the place cell's activity, and therefore depend on space. First, grid cells are driven by the synaptic input provided by the population of place cells. We therefore assume that place cells are the main source of spatial information reaching grid cells, which is in agreement with their development order [3]. Secondly, synapses between place cells and grid cells are subject to a learning process which depends both on pre-synaptic activity, therefore depending on spatial location, and on post-synaptic activity. Plasticity is triggered when the grid cell's activity is above a threshold value. Synaptic efficacies are discrete (*w*_- _<*w*_0_<*w*_+_<*w*_++_) and their change depends on pre-synaptic place cell activity. Four firing rate domains are considered and the synaptic change for each domain, from low to high, is: static, *w*_0_; potentiated *w*_+_; depressed, *w*_-_; potentiated, *w*_++_. When a synapse *w*_+ _is potentiated again its efficacy is raised to *w*_++_.

## Conclusions

**Figure 1:**
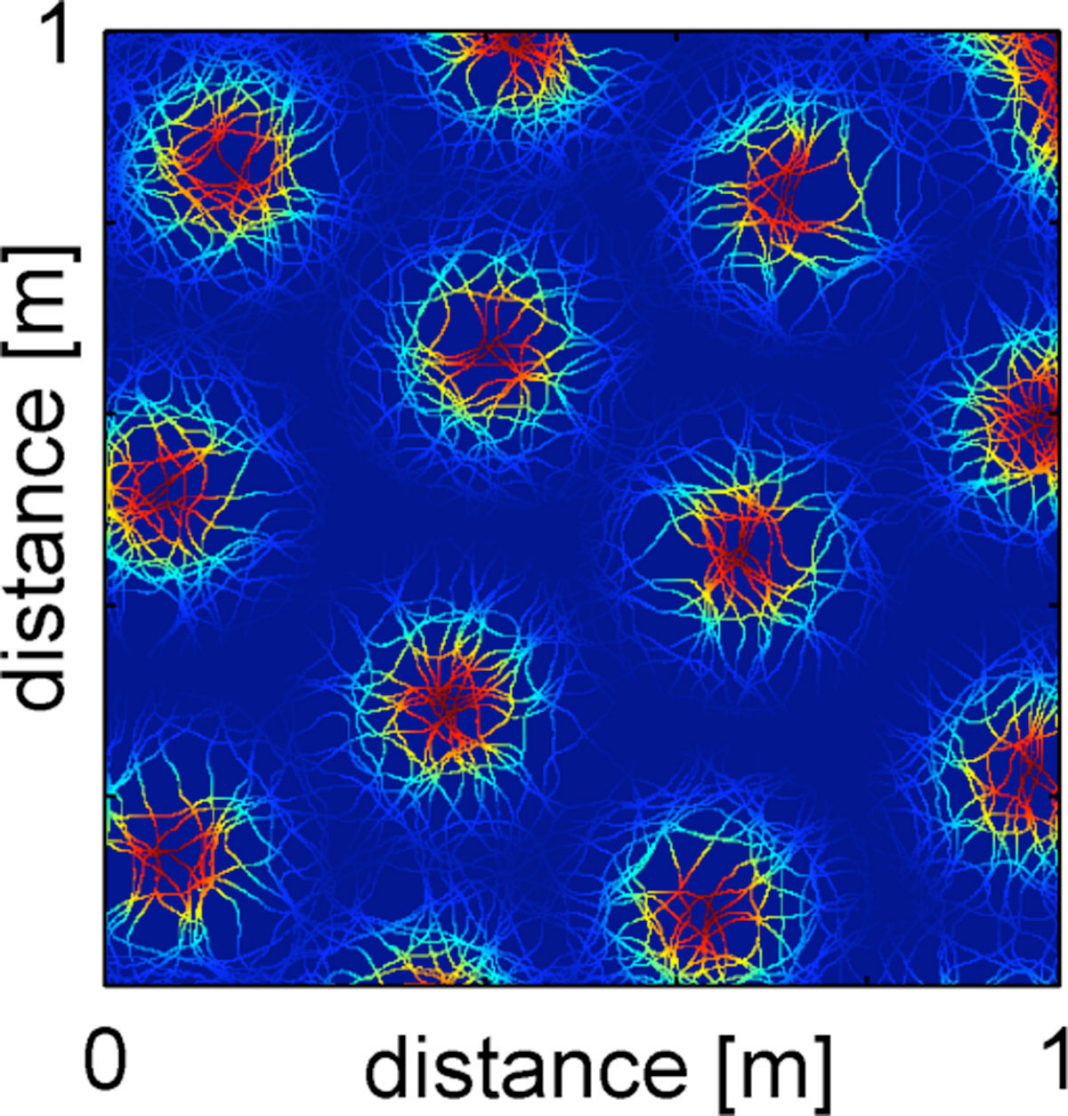
**Average firing rate of a single simulated grid cell as a function of the rat's position (blue to red: 0 to 20 Hz)**.

With the direct input of place cells to grid cells and with the plasticity rule previously described, spatially correlated place cells compete to form the nodes of the grid map. As the animal explores the environment, some place cells are selected over the others to build the grid field. After approximately 30 minutes of exploring randomly an unfamiliar 1 meter square maze, the grid map emerges (see Figure 1). The gridness scores for the resulting grid maps are above 1. The synapses mediating this mechanism can represent the connections between cells in CA1 and the deep layers of mEC. An important feature of this model is that the formation of grid fields is not unique to spatial information: a regular firing rate lattice can be formed from an input population with competing neurons tuned for non-spatial information. Overall, our model is in close agreement with the recently discovered grid cells in primates' visual system [4] and in bat's cortex [5].
